# Excessive Nitrite Affects Zebrafish Valvulogenesis through Yielding Too Much NO Signaling

**DOI:** 10.1371/journal.pone.0092728

**Published:** 2014-03-21

**Authors:** Junbo Li, Wenshuang Jia, Qingshun Zhao

**Affiliations:** Model Animal Research Center, Ministry of Education Key Laboratory of Model Animal for Disease Study, Nanjing University, Nanjing, China; Institute of Cellular and Organismic Biology, Taiwan

## Abstract

Sodium nitrite, a common food additive, exists widely not only in the environment but also in our body. Excessive nitrite causes toxicological effects on human health; however, whether it affects vertebrate heart valve development remains unknown. In vertebrates, developmental defects of cardiac valves usually lead to congenital heart disease. To understand the toxic effects of nitrite on valvulogenesis, we exposed zebrafish embryos with different concentrations of sodium nitrite. Our results showed that sodium nitrite caused developmental defects of zebrafish heart dose dependently. It affected zebrafish heart development starting from 36 hpf (hour post fertilization) when heart initiates looping process. Comprehensive analysis on the embryos at 24 hpf and 48 hpf showed that excessive nitrite did not affect blood circulation, vascular network, myocardium and endocardium development. But development of endocardial cells in atrioventricular canal (AVC) of the embryos at 48 hpf was disrupted by too much nitrite, leading to defective formation of primitive valve leaflets at 76 hpf. Consistently, excessive nitrite diminished expressions of valve progenitor markers including *bmp4*, *has2*, *vcana* and *notch1b* at 48 hpf. Furthermore, 3′, 5′-cyclic guanosine monophosphate (cGMP), downstream of nitric oxide (NO) signaling, was increased its level significantly in the embryos exposed with excessive nitrite and microinjection of soluble guanylate cyclase inhibitor ODQ (1H-[1], [2], [4]Oxadiazolo[4,3-a] quinoxalin-1-one), an antagonist of NO signaling, into nitrite-exposed embryos could partly rescue the cardiac valve malformation. Taken together, our results show that excessive nitrite affects early valve leaflet formation by producing too much NO signaling.

## Introduction

Nitrite, a natural chemical compound, is widely present in the environment and our body. It is a normal part of human diet, found in most vegetables. In the food industry, it is used as a food additive, serving a dual purpose by altering the color of preserved fish and meats and preventing the meats from botulism [1]. Physiologically, it is recognized as an important signaling molecule involved in maintaining perfusion and redox status in tissues, not solely a metabolic product of NO in tissues [2]. It is the main source of NO in hypoxic conditions and oxidative stress states, representing a physiologically critical storage form in blood and tissue [1]. Because of these critical functions, nitrite has been found to be an effective drug as a NO donor to increase blood flow by dilating blood vessels [3], and an intravenous mixture including sodium nitrite has been used as an emergency treatment for cyanide poisoning [5].

However, nitrite is also viewed as a substance that affects our food supply and drinking water adversely. Excessive levels of nitrite in drinking water are found to be associated with illness in newborns and young infants [1]. Infants consuming extra nitrite suffer from methemoglobinemia, a blue baby syndrome with hypoxia, cyanosis and blue color because of nitrite-mediated oxidation of ferric iron in oxyhemoglobin [6]. Additionally, too much nitrite from exogenous sources has been implicated in the development of gastric cancer and other disorders. Therefore, the IARC assigned nitrite as a probable carcinogen (Group 2A) because it can give rise to N-nitroso compounds, potential carcinogens, under conditions in the stomach [7]. Based on these toxic effects, the regulations on the amount of nitrite in our food supply and drinking water are therefore established. For example, the US Environmental Protection Agency suggests that the limitation of human exposure to inorganic nitrite is 1 mg/l [8] and the Joint Food and Agricultural Organization/World Health Organization demands that the acceptable daily intake (ADI) for the nitrite ion is 0.06 mg/kg body weight [9].

Because the amount of chemical compounds containing nitrogen in the environment that are released form modern agriculture and industry has been increased rapidly nowadays, pollutions of nitrite and nitrate have been becoming more and more serious environmental concerns. Though the toxicological mechanisms and health risks of nitrite have been widely investigated [1], [10], little is known whether high level of nitrite is toxic to vertebrate early heart development and performance.

Heart is the first organ to form and play physiological functions during vertebrate embryogenesis. Lining the outflow tract and atrioventricular canal (AVC), cardiac valves play crucial roles in heart function by preventing blood flow retrograde within the heart tube of vertebrates. Because of their complicated structures and long time to form during embryogenesis, valve development is very sensitive to teratogenetic factors. Developmental defects of cardiac valves usually lead to congenital heart disease. The frequency of congenital valve malformations was estimated as high as 5% of live births and 80% of them have been attributed to unknown aetiology [11]. Therefore, uncovering the teratogenetic factors affecting heart valve development would be very helpful to prevent and cure the valve diseases.

In this study, we explore the developmental toxicity of nitrite to vertebrate embryos by exposing zebrafish embryos with different concentrations of sodium nitrite. Our results showed that sodium nitrite caused abnormal heart development of zebrafish embryos in a dose dependent way by directly disrupting formation of atrioventricular valve through producing too much NO signaling.

## Materials and Methods

### Ethics statement

Zebrafish used in this study were housed in the zebrafish facility at Model Animal Research Center (MARC), Nanjing University. The research protocol was approved by IACUC of MARC.

### Exposure of sodium nitrite

Stock solution of 1 g/l sodium nitrite (Purity: 99.99%; Sigma, USA) or sodium chloride (Purity: 99.5%; Sigma, USA) were made with nanopure water (18.2MΩ). Embryos collected from wild type, *Tg(flk1:GFP)* and *Tg(gata1:dsRed)* zebrafish were incubated in the nanopure water containing 60 mg/l of sodium chloride. 1-phenyl-2-thiourea (Sigma, USA) was added to the water in a final concentration of 0.003% at 10 hpf to prevent embryos from developing melanin pigmentation. In exposure experiments, sodium nitrite was added to the water with different concentrations for different times whereas the same amount of sodium chloride was added in control experiments. The water solution were changed every 24 h. To stop exposure, the nitrite-containing medium was replaced with the nanopure water containing 60 mg/l of sodium chloride after the exposed embryos were washed three times. Each exposure experiment was performed 3 times independently. The results were subjected to Student *t*-test. The data were shown as average ± standard errors.

### Live imaging

To observe morphogenetic changes, we anesthetized embryos with ethyl 3-aminobenzoate methanesulfonate (Sigma, USA) for 5 min at room temperature, and then mounted with 3% methyl cellulose (Sigma, USA). Photomicrographs and movies were taken or recorded using Olympus DP71 digital camera (Olympus, Japan) under a fluorescent dissecting microscope (Leica, Germany).

To quantify the toxic effects of sodium nitrite on heart development, we defined EI (edema index), the ratio of the semi-diameter of the pericardial cavity to that of ventricle, to evaluate the defect.

### Histological staining

To observe the histological changes, we fixed zebrafish embryos with 4% paraformaldehyde and embedded in paraffin. The embryos were sectioned consecutively in 5 μm thick. The embryonic tissues were stained with hematoxylin and eosin. Photomicrographs were taken using Olympus DP70 digital camera (Olympus, Japan) under a polarizing microscope Olympus BX51 (Olympus, Japan). At least three consecutive sections were observed to make sure the section containing the embryonic heart structures of AVC, and/or leaflets of atrioventricular valve.

### Whole mount *in situ* hybridizations

Whole mount *in situ* hybridizations to detect mRNA messages were performed as described previously [12]. The templates for anti-sense RNA probes including *amhc* (NM_198823), *bmp4* (NM_131342), *cmlc2* (NM_131329), *has2* (NM_153650), *klf2a* (NM_131856), *notch1b* (NM_131302), *nppa* (NM_198800) and *vmhc* (NM_001112733) were cloned from zebrafish cDNAs using primer sets of AAGCATTCGCTCGTGGACT and CATCCAGTGTCTGCTGGT (for *amhc*), TGCCAAGTCCTACTGGGAG and CGTGATTGGTGGAGTTGAG (for *bmp4*), CTCTTCCAATGTCTTCTCC and TATTTCCAGCCACGTCTA (for *cmlc2*), ACGACACTGTTCGGCATTT and CAGCGGGTTTGTTGGTTG (for *has2*), GGCCAAACATGTGAGGTG and GCTGTATCTTGTGCCGCT (for *notch1b*), ATGGCCGGGGGACTAATTC and CCGCGTATTGCAGCTAACC (for *nppa*), CGTATTTCCTCCGCTTCTTA and TTTCCAGAGTCCGTTCCTAC (for *klf2a*), and CTCCTGGTGCAAAGAATC and TTCAGCTCAGAGTGGCATTCGTCC (for *vmhc*), respectively.

Whole mount immunohistochemistry to detect Dm-grasp was performed using primary antibody ZN-5 (Zebrafish International Resource Center, USA) and secondary antibody anti-Mouse IgG (H+L) (Jackson Lab, USA). Briefly, embryos were fixed with 4% PFA for 90 min, washed with PBS-Triton (1×PBS, 0.3% Triton-100) for 5 minutes 3 times, blocked with blocking solution (2% Blocking Reagent (Roche, Switzerland) and 10% sheep serum in PBS-Triton) for 1-4 hr at room temperature. The embryos were then incubated with ZN-5 (1∶500) overnight at 4°C. To remove the nonspecific binding of the antibody, embryos were washed with PBS-Triton 8 times, each for 30 minutes. After washing, the embryos were incubated with anti-Mouse IgG (1∶500) overnight at 4°C. To remove the nonspecific binding of IgG, embryos were washed with PBS-Triton 3 times, each for 30 min. The embryos were then mounted in 1% low melting agarose. Photomicrographs were taken using Olympus DP71 digital camera (Olympus, Japan) under a fluorescent dissecting microscope (Leica, Germany).

### Measurement of cyclic GMP in zebrafish embryos

The amount of cGMP in zebrafish embryos at different developmental stages were determined by competitive ELISA assay using Cyclic GMP EIA Kit (Cayman Chemical, USA). Before measurement, embryonic samples and a standard curve of cGMP amount were prepared in a way of no acetylation by following the manufacture's instruction.

To prepare the samples, we homogenized 200 zebrafish embryos at each developmental stage with 2 ml of 5% trichloroacetic acid (TCA) on ice. The solution was subjected to centrifugation at 1,500 g for 10 min. The supernatant was carefully transferred to a clean test tube and mixed with five volumes of ether for 10 s. After mixture, the organic and aqueous phase was allowed to separate. The ether layer was carefully removed and the extraction was repeated two more times. The aqueous layer was finally heated to 70°C for 5 min to remove the residual ether. It was then ready for use to measure cGMP amount.

To prepare the standard curve, we diluted the cGMP EIA standard with EIA buffer following the manufacturer's instruction. We then performed the assay by adding the standard aliquots, prepared samples with two dilutions (1/2 dilution) with three repeats and other reagents including antibody sequentially to the plate provided in the Kit. The plate was covered with plastic film and incubated at 4°C for 18 hr. After incubation, the plate was developed with Ellman's Reagent in the dark at 4°C for 2 hr. The plate was then read at wavelength of 412 nM using Sunrise Remote/Touch Screen (TECAN, Austria). A standard curve of cGMP amount was subjected to plot following the manufacture's instruction using on-line software (http://www.myassays.com/assay.aspx?id=394). The amount of cGMP in each sample was calculated from the standard curve. The experiment was performed twice independently. The values of cGMP amount in all the control embryos were normalized to 1.0. The relative amount of cGMP level in the nitrite-exposed embryos was the valve of the amount of treated embryos divided by that of control embryos in the same developmental stage. The data were shown as average ± standard errors. The results were subjected to Student *t*-test.

### Measurement of nitrite level in zebrafish embryos

The amount of nitrite in zebrafish embryos was determined by using Griess Reagent (Sigma, USA). To measure the nitrite level, 100 embryos at each stage were used. They were dechorionated, pooled and then weighed by an electronic analytical balance. After being weighted, the embryos were washed with nanopure water, homogenized using 500 μl nanopure water, followed by centrifuging briefly. The supernatant was then mixed with 1× Griess reagent in a ratio of 1∶1. After incubation for 15 min, the absorbance at 540 nm of each sample was read with a spectrometer (Eppendorf, Germany).

Standard curve defining the relationship between amount of nitrite and the absorbance was plotted using sodium nitrite with known concentration following the instructions of the Kit. Briefly, absorbance at 540 nm was read after 15 minutes incubation with 1×Griess Reagent. Griess Reagent only and nanopure water only were used as negative control, respectively. Standard curve was plotted with the concentration of nitrite sodium ranging from 0.05 mg/l to 25 mg/l in the X-axis and absorbance in the Y-axis. The linear equation was established with R^2^>0.99. The concentration of each sample was calculated from the equation of the standard curve based on its absorbance.

### Microinjection of ODQ into zebrafish embryos

To rescue the developmental defects of the embryo hearts caused by excessive sodium nitrite, 1 nl of 50 nM ODQ dissolved in dimethyl sulfoxide (Sigma, USA) were microinjected into the yolk sac close to zebrafish embryonic heart at 30 hpf. As to the embryos observed at 108 hpf, the microinjection was repeated on the embryos at 54 hpf, and 78 hpf, respectively.

## Results

### Nitrite exposure causes abnormal cardiac development of zebrafish embryos in a dose dependent manner

**Figure 1:**
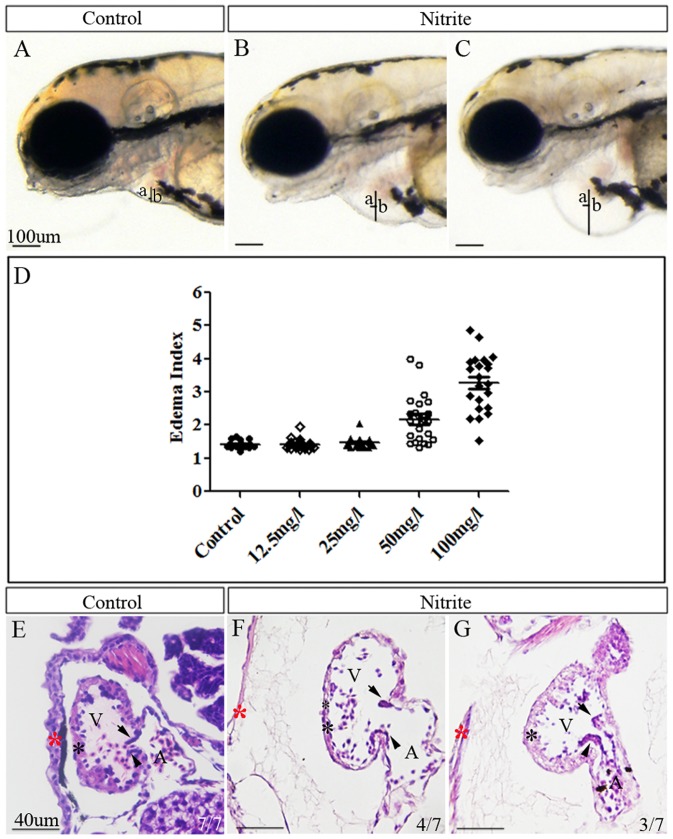
Sodium nitrite caused defective development of zebrafish heart in a dose dependent way. (A–C) Zebrafish embryos exposed to 100 mg/l sodium nitrite from 10 hpf exhibited cardiac edema from slight phenotype (B) to severe phenotype (C) compared to control embryos with normal heart (A) at 108 hpf. (D) A scatter plot showing the edema index (EI) of embryos exposed with different concentration of sodium nitrite. EI is defined as b/a. Average ± standard errors of EIs were shown in lines. (E–G) Histological sections showing 100 mg/l sodium nitrite exposure caused defective structure of zebrafish heart at 108 hpf. Compared to control embryos with normal pericardial membrane, myocardium and both superior and inferior valve leaflets (E), 4/7 embryos exposed to the nitrite showed thinner pericardial membrane and myocardium, and only superior valve leaflet but no formation of inferior valve leaflet (F), whereas 3/7 of the treated embryos displayed thinner pericardial membrane and myocardium and no formation of either superior or inferior leaflets (G). a: the semi-diameter of ventricle; b: the semi-diameter of the pericardial cavity. Red star (*): pericardial membrane; Black star (*): myocardium; Black arrow: position of superior valve leaflet; Black arrowhead: position of inferior valve leaflet; A: atria; V: ventricle.

To understand the toxic effects of nitrite on embryogenesis, we exposed zebrafish embryos to sodium nitrite with different concentrations. In our pioneer experiment, no defective phenotypes were found in the 108 hpf embryos exposed with 100 mg/l sodium nitrite from 0 to 10 hpf. We therefore treated zebrafish embryos starting from 10 hpf with different concentrations of sodium nitrite. Generally, the morphology of the embryos exposed to 100 mg/l sodium nitrite looked pretty normal till 48 hpf though they showed slightly shorter body lengths at 48 hpf (Figure S1 A–D). At 76 hpf, the treated embryos still displayed normal diameter of eye, vertical diameter of head, and heart development when compared to control embryos (Figure S1E–G). However, the lengths of the exposed embryos were significantly shorter (P<0.05) than those of control embryos (Figure S1E–G). Additionally, the nitrite-treated embryos exhibited no response to the mechanical stimulus whereas the control embryos escaped rapidly by swimming away when the mechanical stimulus was administrated (Movie S1, S2). The results suggest that excessive nitrite affect the development of nerve system or muscle. At 84 hpf, some embryos treated with sodium nitrite exhibited pericardial edema (Figure S1H–I). The pericardial edema became more severe when the embryos reached 108 hpf (Figure 1B–C).

To quantify the severity of the pericardial edema caused by sodium nitrite exposure, we used edema index (EI) to describe severity of the edema (Figure 1A–C). As shown in Figure 1D, the EIs of control embryos, 12.5, 25, 50 and 100 mg/l nitrite-treated embryos at 108 hpf were 1.42±0.16 (n = 18), 1.42±0.15 (n = 24), 1.47±0.17 (n = 20), 2.18±0.72 (n = 23), and 3.28±0.86 (n = 22), respectively. Statistical analysis demonstrated that EIs of 12.5 mg/l group or 25 mg/l group were similar to those of the control group (p>0.05). However, the EI of 50 mg/l group was significantly higher than that of the control group (p<0.01) whereas the EI of 100 mg/l group was significantly bigger than that of the 50 mg/l group (p<0.01). Morphological observation revealed that 56.5% (13/23) of the embryos from 50 mg/l group exhibited significantly edema phenotype whereas 95.4% (21/22) of the embryos from 100 mg/l group displayed significant edema (EI≥2.00; Figure 1D). 45.6% (10/22) of the embryos from 100 mg/l group had an EI larger than 3.5 whereas only 8.7% (2/23) of the embryos from 50 mg/l group had an EI larger than 3.5. Taken together, these results suggested that sodium nitrite caused abnormal heart development of zebrafish embryos in a dose dependent manner.

In order to know the teratological effects of nitrite on zebrafish embryos at histological level, we observed the sections of the 100 mg/l sodium nitrite exposed embryos with EI larger than 2.0 at 108 hpf. Compared to control embryos (Figure 1E), the nitrite-exposed ones exhibited significantly thinner pericardial membrane and myocardium though the myocardium in the exposed embryos thinned in different extents (Figure 1F–G). Additionally, the nitrite-exposed embryos displayed deformed cardiac valves (Figure 1F–G). In the control embryos at 108 hpf (Figure 1E), both superior and inferior valve leaflets were formed to extend into the ventricular lumen [13]. However, the nitrite-exposed embryos had no normal valve leaflets formed yet (Figure 1F–G). Because the presence of valves between ventricle and atria prevents blood from flowing back from the ventricle to atria when ventricle contracts, the defective valves suggest that the embryos might exhibit an abnormal phenotype with a retrograde blood flow. To confirm this defect, we examined the blood flow in the embryos at 108 hpf. Unlike control embryos that had a normal blood flow (Movie S3), we found an obvious retrograde blood flow occurring in the nitrite-exposed embryos at 108 hpf (Movie S4).

### Continuous exposure to sodium nitrite after 36 hpf is responsible for the abnormal heart development of zebrafish embryos

**Figure 2:**
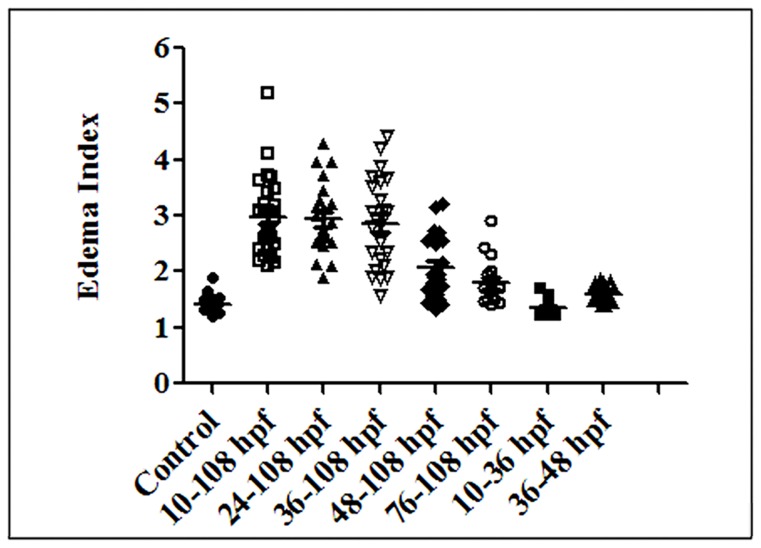
A scatter plot showing that sodium nitrite affected zebrafish heart development starting from 36 hpf. Nitrite-exposed embryos were treated with 100 mg/l sodium nitrite for different time window shown in X-axis. EI (shown in Y-axis) of each embryos was measured and shown in the plot. Average ± standard errors of EIs were shown in lines.

To determine when nitrite plays its toxic effects on zebrafish heart development, we exposed zebrafish embryos with 100 mg/l of nitrite from different developmental stages for different time. As shown in Figure 2, the zebrafish embryos exposed to nitrite from 24–108 hpf (EI = 2.94±0.63, n = 23) and 36–108 hpf (EI = 2.86±0.82, n = 23) both had a similar EI (p>0.05) to that of the embryos exposed to nitrite from 10–108 hpf (EI = 2.99±0.68, n = 28). However, the EI of embryos exposed to nitrite from 76–108 hpf (EI = 1.75±0.41, n = 19) was smaller than (p<0.05) that of embryos exposed to nitrite from 48–108 hpf (EI = 2.07±0.58, n = 22), and both were significantly smaller (p<0.01) than that of the embryos exposed to nitrite from 10–108 hpf. Consistent with the results, the embryos exposed to nitrite from 10–36 hpf (EI = 1.36±0.12, n = 21) displayed similar EI to that of control embryos (EI = 1.42±0.16, n = 18) (p>0.05), and the EI of the embryos exposed to nitrite from 36–48 hpf (EI = 1.61±0.11, n = 16) was bigger (p<0.01) than control embryos, smaller than (p<0.01) that of the embryos treated with nitrite from 48–108 hpf, but similar to (p>0.05) that of the embryos treated with nitrite from 76–108 hpf. Taken together, our results suggested that exposure of excessive nitrite before 36 hpf had no toxic effect on zebrafish heart development and the continuous exposure of nitrite after 36 hpf was responsible for the abnormal heart development of zebrafish embryos.

### Overdosing nitrite impairs zebrafish AV canal formation as early as 43hpf

**Figure 3:**
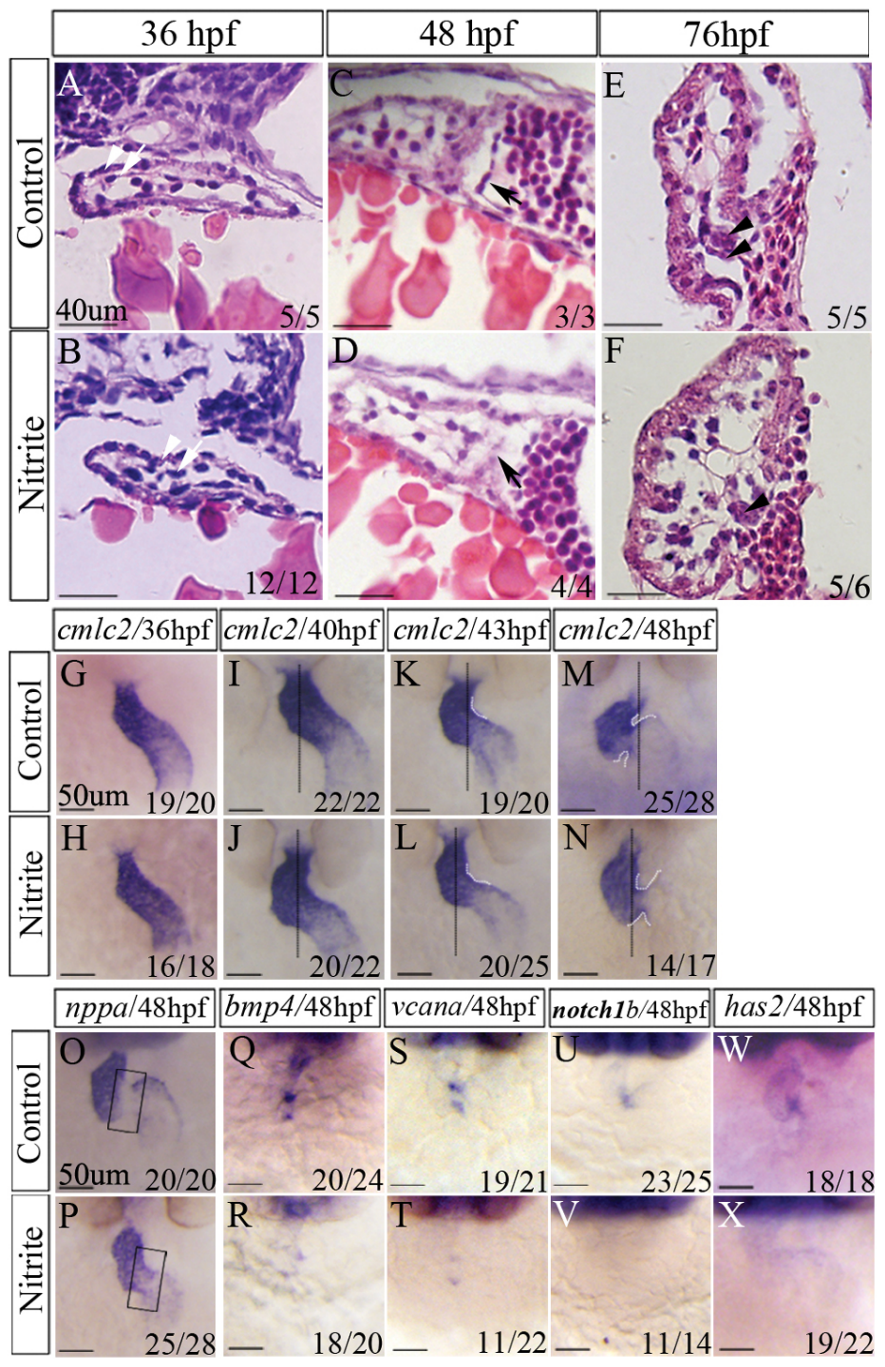
Histological and molecular analyses revealing excessive nitrite affected zebrafish heart valve development directly. Nitrite-exposed embryos were treated with 100 mg/l sodium nitrite from 10 hpf. (A–F) Histological sections showing defective valve development caused by nitrite exposure occurred from 48 hpf. At 36 hpf, the embryos exposed to nitrite exhibited similar histological heart structure (B) to that of control zebrafish, comprising one layer of myocardium and one layer of endocardium (A). At 48 hpf, endocardial cells of control embryos exhibited cuboidal shape in AVC between two chambers (C) but the exposed embryos did not have cuboidal endocardial cells in AVC (D). At 76 hpf, invagination of endocardial cells into cardiac jelly in AVC in control embryos formed superior primitive valve leaflet consisting of multilayer of cells (E); however, no superior primitive valve leaflet (no multilayer cells in the superior part of AVC) was formed in nitrite-exposed embryos (F). (G–N) Cardiac looping in zebrafish embryos shown by the expression of *cmlc2* revealing abnormal cardiac looping caused by nitrite exposure occurred as early as 43 hpf. The expression pattern of *cmlc2* was not affected by nitrite exposure at 36 hpf (G–H) and 40 hpf (I–J), but slightly abnormal (shown in white dotted curve) at 43 hpf (K–L) and obviously abnormal (shown in white dotted curve) at 48 hpf (M–N). (O–X) Nitrite exposures altered the expressions of molecular makers of valve progenitors at 48 hpf. *nppa* was not expressed in the AVC (rectangular box) of control embryos (O) but was ectopically expressed in the AVC of nitrite-exposed embryos (P). Compared to control embryos, nitrite exposure significantly decreased or abolished expressions of *bmp4* (Q, R), *vcana* (S, T), *notch1b* (U, V) and *has2* (W, X) in AVC. The number shown in the lower right-hand corner was the number of embryos exhibiting the typical phenotype shown in the panel to the number of embryos totally observed. Black arrow: position of cuboidal endocardial cells; Black arrowhead: position of superior primitive leaflet; White arrow: endocardium; White arrowhead: myocardium.

To reveal how the zebrafish heart was affected by excessive nitrite, we examined the histological and molecular changes of nitrite-exposed embryos (treated by 100 mg/l nitrite from 10 hpf) at 36, 48 and 76 hpf, respectively. As shown in Figure 3, no histological difference was found between the hearts of control embryos and those of nitrite-exposed embryos at 36 hpf (Figure 3A–B). Like the control embryos, the nitrite-exposed embryos exhibited a single layer of myocardium lined by a single layer of squamous endocardial cells (endocardium). At 48 hpf, the nitrite-exposed embryos showed normal myocardium and endocardium in atria and ventricle (Figure 3C–D). However, unlike the control embryos which had cuboidal endocardial cells lining the AV canal (Figure 3C), the nitrite-exposed embryos at 48 hpf lacked the remodeled cuboidal cells (Figure 3D). When examined at 76 hpf, both control and nitrite-exposed embryos had normal developed atria and ventricle (Figure 3E–F). However, unlike the control embryos displaying their normal superior primitive valve leaflet and inferior AVC structure respectively (Figure 3E), the nitrite-exposed embryos showed obvious defective development of both superior and inferior AVC structure (Figure 3F).

To further confirm the findings, we analyzed the expression patterns of myocardial and endocardial specific genes in the developing heart of nitrite-exposed embryos. It is known that *amhc* encodes a gene specifically expressed in atrial myocardiocytes [14] and *vmhc* encodes a gene specific expressed in ventricular myocardiocytes [15]. Performing whole mount *in situ* hybridization, we found that the expression patterns of the two genes were not altered in the nitrite-exposed embryos when compared to control embryos at 36, 48 and 76 hpf, respectively (Figure S2A-L). Similarly, *flk1*, the endothelial marker gene [16], was expressed normally in atria and ventricle of the nitrite-exposed embryos at 36, 48 and 76 hpf, respectively (Figure S2M-R, M′-R′). However, its expression in the region of AVC exhibited some reduced level in the nitrite-exposed embryos at 76 hpf (Figure S2R, R′). To confirm the observation, we used the ZN5 monoclonal antibody that recognizes Dm-grasp, a cell surface adhesion molecule of the immunoglobulin superfamily [17], to visualize the differentiated endocardial cells in AVC [18]. The results showed that nitrite-exposed embryos at 76 hpf lacked the differentiated endocardial cells in AVC (Figure S2S-T, S′-T′). Taken together, our results were consistent with the histological observation that nitrite treatment did not affect the development of atria and ventricle but affected the formation of AV canal.

To verify the changes of AV canal formation in nitrite-exposed embryos, we examined the expression of *cmlc2*, a gene encoding cardiac sarcomere proteins that is expressed in differentiating myocardiocytes and is essential for the formation of AVC [15], [19]. When examined at 36 hpf, the expression of *cmlc2* was pretty normal in the nitrite-exposed embryos (Figure 3G–H). The expression pattern became obviously abnormal in the region (abnormal looping) between atria and ventricle when the nitrite-exposed embryos reached 48 hpf (Figure 3M–N). Detailed analysis revealed the looping defect first occurred in the embryos at 43 hpf (Figure 3K–L). Because the region between atria and ventricle is the place where AVC gives rise to AV valves, the abnormal looping suggested the progenitors of AV valves could be affected by excessive nitrite. Consistently, *nppa* (natriuretic peptide precursor type A), normally expressed in myocardial cells of ventricle and atria and absent from the AVC [20], [21] (Figure 3O), was ectopically expressed in the region between atria and ventricle besides its normal expression domains in the nitrite-exposed embryos (Figure 3P).

To further confirm the defective AVC formation, we examined the expressions of the AV valve maker genes in nitrite-exposed embryos. Normally, *bmp4*, a secreted growth factor of the bone morphogenetic protein family (Figure 3Q), and *vcana* (previously known as *versican*) (Figure 3S) are restricted to express in the myocardium [19], [22] whereas *notch1b*, zebrafish *notch* homolog (Figure 3U) and *has2* (Figure 3W) are restricted to the endocardium [22], [23] of AVC of the embryos at 48 hpf. However, expressions of all these AV canal marker genes were significantly reduced (*bmp4* and *vcana*) and or even lost (*notch1b* and *has2*) in the nitrite-exposed embryos at 48 hpf (Figure 3Q–X). Time window analyses revealed that the reduced or lost expressions occurred in the nitrite-exposed embryo as early as 43 hpf (Figure S3). Taken together, the results suggested that excessive nitrite could affect AVC formation as early as 43 hpf.

### Reducing NO signaling can rescue the abnormal heart defect caused by excessive nitrite

It is known that changes of hemodynamics cause defective valvuogenesis [24]. To investigate whether the valve defect in the nitrite-exposed embryos results from changes of blood flow after nitrite exposure or not, we first examined the development of blood cells and vessels. Analyses on the fluorescent imaging of embryos produced from *Tg(gata1:dsRed)* and *Tg(flk1:GFP)* at 24, 36 and 48 hpf revealed that the nitrite-exposed embryos had normal development of red blood cells (Figure S4A–L) and vessels (Figure S4O-T, O′-T′), respectively. Consistently, *klf2a*, normally expressed in the valve precursors in response to reversing flows [25], did not change its expression pattern after nitrite exposure (Figure S4M–N). The results demonstrated that the valve defect in the embryos exposed with excessive nitrite was not due to hemodynamic changes.

Previously, it was demonstrated that fish uptake nitrite from ambient water directly and accumulate it in its body [26], [27]. To uncover how nitrite affected zebrafish heart valve development, we checked nitrite amount in zebrafish embryos before and after sodium nitrite exposure by using Griess Reagent (Sigma, USA). The results showed that the amount of nitrite was 1.1 mg/kg embryos at 24 hpf, 0.9 mg/kg embryos at 36hpf, 3.0 mg/kg embryos at 48 hpf and 13.3 mg/kg embryos at 76 hpf, respectively. In contrast, it was increased to 43.0 mg/kg embryos at 24 hpf, 166.1 mg/kg embryos at 36hpf, 285.1 mg/kg embryos at 48 hpf and 141.3 mg/kg embryos at 76 hpf after the embryos were exposed to 100 mg/l nitrite sodium, respectively (Figure S5).

**Figure 4:**
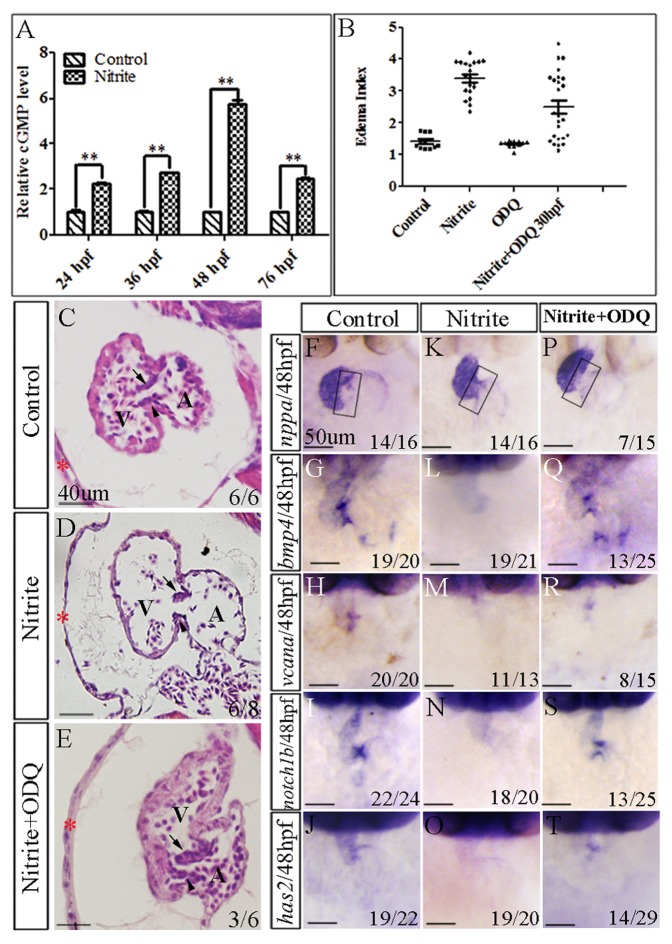
Inhibiting NO signaling in nitrite-exposed embryos partially rescued defective development of cardiac valve in zebrafish embryos. (A) The cGMP level was dramatically increased in the nitrite-exposed embryos at 24, 36, 48 and 76 hpf, respectively. **: P<0.01. The values of cGMP amount in all the control embryos were normalized to 1.0, respectively. The value of cGMP amount in the nitrite-exposed embryos was the fold of the control embryos at the same developmental stage. (B) A scatter plot showing the increased EIs in the nitrite-exposed embryos were significantly reduced by microinjecting ODQ (sGC inhibitor) into nitrite-exposed embryos. Different treatments of embryos were shown in X-axis. EI (shown in Y-axis) of each embryos was shown in the plot. Average ± standard errors of EIs were shown in lines. (C–E) Defective histological structures of heart caused by excessive nitrite were partially rescued by microinjection of ODQ into nitrite-exposed embryos. Embryos were observed at 108 hpf. Compared to control embryos with normal pericardial membrane, myocardium and both superior and inferior valve leaflets (C), 6/8 embryos exposed to the nitrite showed thinner myocardium and defective formation of either superior or inferior leaflets (D). ODQ microinjection resulted in 3/6 of the embryos developed both superior and inferior leaflets (E). (F–T) Microinjection of ODQ partially rescued the diminished expressions of valve progenitor makers in zebrafish embryos at 48 hpf. *nppa* is not expressed in the AVC (rectangular box) of control embryos (F) but was ectopically expressed in the AVC of nitrite-exposed embryos (K). Microinjection of ODQ into nitrite-exposed embryos prevented 7 of 15 embryos from expressing *nppa* in AVC (P). Compared to control embryos, nitrite exposure significantly decreased or abolished expressions of *bmp4* (G, L), *vcana* (H, M), *notch1b* (I, N) and *has2* (J, O) in AVC. However, microinjection of ODQ into nitrite-exposed embryos resumed expressions of *bmp4* (Q), *vcana* (R), *notch1b* (S) and *has2* (T) in about half embryos. Red star (*): pericardial membrane; Black arrow: position of superior valve leaflet; Black arrowhead: position of inferior valve leaflet; A: atria; V: ventricle.

Because excess NO has been found to form in the body of nitrite-exposed adult zebrafish [27], we therefore tested whether the nitrite exposure could elevate NO signaling in the exposed zebrafish embryos. To do this, we measured the cGMP level, the downstream signal of NO signaling, in the embryos. As shown in Figure 4A, the cGMP level was significantly increased in the nitrite-exposed embryos (100 mg/l of sodium nitrite treated from 10 hpf) at 24, 36, 48 and 76 hpf, respectively (Figure 4A). The result was consistent with the previous report that nitrite readily affects cGMP production [28] and suggested that NO signaling was increased in the embryos exposed with excessive nitrite.

To determine whether the elevated NO signaling was responsible for the defective phenotype resulting from excessive nitrite, we microinjected ODQ, an inhibitor of soluble guanylate cyclase (sGC) and an antagonist of NO signaling to block the production of cGMP [29], to the nitrite-exposed embryos, and then observed their heart development at 108 hpf. As shown in Figure 4B, the embryos microinjected with ODQ displayed a similar (p>0.05) EI (EI = 1.33±0.10, n = 12) to that of control embryos (EI = 1.41±0.21, n = 12) without the microinjection. However, the nitrite-exposed embryos microinjected with ODQ displayed a significantly smaller (p<0.01) EI (EI = 2.50±1.05, n = 25) than nitrite-exposed embryos (EI = 3.39±0.54, n = 20) without the microinjection (Figure 4B). Among 25 embryos, 9 (36%) had a normal EI to that of control embryos (EI≤1.62) and 15 (60%) embryos had bigger EI (≥2.0) while all of the un-microinjected nitrite-exposed embryos had an EI bigger than 2.0. The results suggested that microinjection of ODQ rescued, at least partly, the developmental defect of heart resulting from excessive nitrite.

To confirm the rescue, we sectioned the embryos with EI close to normal ones at 108 hpf. The results showed the nitrite-exposed embryos microinjected with ODQ displayed similar thickness of pericardial membrane and myocardium to the control embryos (Figure 4C, 4E). Additionally, 50% (3/6) of the nitrite-exposed embryos microinjected with ODQ showed normal formation of both superior and inferior leaflets at AV canal (Figure 4E) whereas 75% (6/8) of nitrite-exposed embryos showed defective leaflet formation (Fig 4D). Moreover, whole mount *in situ* hybridization analyses revealed that the abnormal expression of *nppa* (Figure 4K) was no longer present in AVC in part of the nitrite-exposed embryos that were microinjected with ODQ (Figure 4P). Furthermore, the reduced or depleted expressions of *bmp4*, *vcana*, *notch1b* and *has2* in the nitrite-exposed embryos were resumed to normal level in around half of the nitrite-exposed embryos that were microinjected with ODQ (Figure 4G–J, L–O, Q–T).

Taken together, our results strongly suggested that excessive nitrite caused abnormal development of zebrafish heart through directly affecting AV valve formation by yielding too much NO signaling.

## Discussion

Although zebrafish cardiac valves are structurally smaller and simpler than those in amniotes, the mechanisms underlying their formation are highly conserved. Therefore, zebrafish provides an excellent model addressing underlying mechanism of heart valve formation and the roles of environmental factors in affecting valvulogenesis of amniotes [30]. Nitrite, a natural inorganic chemical that is widely present in the environment, had been recognized as an inert substance for a long time. However, recent researches demonstrate that it plays crucial roles in our body by maintaining the homeostasis of nitrate-nitrite-NO pathway [1], [28], [31]. In our body, about 50% of nitrite is formed endogenously from NO, which itself is mainly generated by endothelial nitric oxide synthase (eNOS) using L-arginine as a substrate and the other is obtained exogenously by daily diet and reduction of salivary nitrate [1], [32], [33]. Therefore, the amount of nitrite and nitrate in the environment largely affects the concentration of nitrite in our body and extra amount of nitrite than necessary would be a toxic compound by disrupting the homeostasis. Actually, excessive nitrite taken from exogenous sources into human body caused a variety of diseases such as methemoglobinemia, gastric cancer and other disorders or even death [1], [34]. In this study, we found excessive nitrite caused abnormal development of zebrafish heart in a dose dependent way besides shortened body length and defective development of nerve system or muscle (Figure 1, Figure 2, and Figure S1). Performing analyses on the embryos exposed with excessive nitrite at histological, cellular and molecular levels (Figure 3, Figure 4, Figure S2, Figure S4), we demonstrated that the abnormal heart development were due to defective formation of the AV valve.

Development of the valve structures is controlled by a regulatory network comprising multiple signaling such as BMP and Tbx sent from myocardium, and VEGF, Notch, and Wnt derived from endocardium [35]. Dysfunction of these signaling leads to malformation of valve structures, resulting in cardiac edema in zebrafish embryos. Treating the embryos with excessive nitrite, we showed that expressions of valve progenitor markers including *bmp4*, *has2*, *vcana* and *notch1b* were all diminished in the embryos at 48 hpf (Figure 3, Figure S3). The results suggest that excessive nitrite affects zebrafish AV valve development by disrupting the formation of valve progenitors.

Nitrite is demonstrated to function as a reservoir of NO signaling in mammals. It can readily be reduced to NO under certain physiological and pathological conditions in response to oxygen levels and oxidative stress in the tissue environment by a number of nitrite reductases, such as deoxyhemoglobin, myoglobin, neuroglobin, cytoglobin, xanthine oxidase, cytochrome c, and aldehyde dehydrogenase 2, and also by acidic environment [1]. Taking up from ambient water, fish are able to accumulate nitrite in their bodies and reduce it to NO mainly by deoxygenated heme groups inside red blood cells. Therefore, excess NO is formed in the body of adult zebrafish exposed with excessive nitrite [27]. Consistent with the discoveries, we found that nitrite was accumulated and that the amount of cGMP, the second messenger of NO signaling, was greatly increased in the embryos exposed with excessive nitrite (Figure 4, Figure S5). Reducing NO signaling by ODQ, an inhibitor of sGC, could partially resume expressions of the valve progenitor markers that were diminished in the nitrite-exposed embryos at 48 hpf (Figure 4), resulting in partially rescue of the abnormal AV valve defects and cardiac edema (Figure 4). It is predictable that agonist of NO signaling could mimic the excessive nitrite to cause abnormal cardiac valve development in zebrafish embryos. Taken together, the results strongly suggest that excessive nitrite affects early valve leaflet formation at least partially by producing too much NO signaling.

NO signaling is required for normal embryonic development. It plays a crucial role in cardiac valve formation [36] by activating sGC heterodimer consisting of one A subunit (GUCY1A2 or GUCY1A3) and one B subunit (GUCY1B3), leading to production of cGMP to promote endothelial cell survival and migration [37]. Inhibition of sGC blocks endothelial-mesenchymal transition (EndMT) in AVC explants and decreases cushion cellularization in mouse at E9.5 and E10.5 [38]. As a result, mice with homozygous eNOS knockout exhibit defective cardiac valves [38]. However, too much NO signaling is associated with early embryo loss [39]. In nitrite-exposed fish, the massively produced NO is responsible for part of the toxic action of nitrite at high concentrations [27]. In this study, we showed that too much NO signaling, elicited from the excessive nitrite and marked by greatly increased cGMP level, is detrimental to heart valve development of vertebrates.

Notch signaling is known to regulate cardiac valve development by activating the NO-sGC axis through an autocrine loop [37]. Human with NOTCH1 mutations suffered from defective cardiac valves [40]. In mouse embryos, Notch signaling increases NO production simultaneously with induction of the NO receptor sGC including *Gucy1a3* and *Gucy1b3* by binding RBPJ to their promoters [37]. Inhibition of Notch signaling specifically in endocardial cells *in vivo* blocks EndMT and decreases *Gucy1b3* expression, leading to developmental defect of cardiac valve [37]. In zebrafish, *notch1b* is restricted to endocardium of AVC [22]. Knocking down Notch signaling in zebrafish results in disruption of cardiac valve formation [18]. In this study, we found too much NO signaling diminished *notch1b* expression and inhibiting the excessive NO signaling could rescue *notch1b* expression, leading to rescue defective cardiac valve development in zebrafish embryos (Figure 4). The results suggest a negative feedback loop between Notch signaling and NO signaling is present in vertebrate valvulogenesis.

In addition to reduction to NO, nitrite itself can directly regulate gene functions in a NO-independent manner through heme nitrosylation- and thiol nitrosation-based mechanisms. It efficiently increases tissue cGMP levels by NO-independent nitrosylation of sGC and up-regulates the expression of Hsp70 [28] that is an endogenous sGC activator working with other factors to increase cGMP levels [41]. These activities are consistent with the known profile of NO action [28] and likely occurred in the nitrite-exposed zebrafish embryos because L-NAME (N^G^-nitro-L-arginine methyl ester), an inhibitor of NOS, were much less efficient in rescuing the zebrafish heart valve defects caused by excessive nitrite exposure than ODQ (data not shown).

Other than cGMP-dependent action, nitrite may affect gene expression in a cGMP-independent manner by means of post-translational oxidative and/or nitrosative modification of transcription factors including NF-κB, AP-1, and p53 [42]. In this study, we did not examine expression changes of the genes, but the post-translational oxidative and/or nitrosative modification of transcription factors by nitrite might be the explanation why inhibiting cGMP production only partially rescued the cardiac valve defects caused by excessive nitrite.

## Supporting Information

Figure S1 Morphological changes of nitrite-exposed embryos at early development. Nitrite-exposed embryos were treated with 100 mg/l sodium nitrite from 10 hpf. They displayed a similar morphological phenotype to control embryos at 24 hpf (A, B). At 48 hpf, the nitrite-exposed embryos (D) looked very similar to control embryos (C) except that they showed a slightly shortened body length (D). When reaching 76 hpf, the nitrite-exposed embryos displayed normal diameter of eye, and heart development (E, G) when compared to control embryos (F, G). However, the body length of the exposed embryos was significantly shorter than control embryos (F, G). At 84 hpf, some of nitrite-exposed embryos started to exhibit cardiac edema (H, I). *, P<0.05.
(TIF)

Figure S2 Excessive nitrite exposure affected endothelial cell accumulation and differentiation in the AVC at 76 hpf. Nitrite-exposed embryos were treated with 100 mg/l sodium nitrite from 10 hpf. Expression patterns of *vmhc* in ventricle and *amhc* in atria of nitrite-exposed embryos were similar to those in control embryos at 36 (A, B; G, H), 48 (C, D; I, J) and 76 hpf (E, F; K, L), respectively. Excessive nitrite exposure did not change the distribution of endothelial cell in the AVC of embryos at 36 hpf (M-N, M′-N′) and 48 hpf (O-P, O′-P′). Compared to control embryos at 76 hpf (Q-S; Q′-S′), 5/8 nitrite-treated embryos had fewer endothelial cells (R, R′) and 14/15 nitrite-exposed embryos lost the expression of Dm-grasp (T, T′), maker of endothelial cell differentiation in AVC, at the same stage. Panel M′-T′ were the magnification of the region outlined by rectangle in Panel M-T, respectively. Heart was outlined by dot-lined curves and the AVC in Panel M′-T′ was outlined by a small rectangle.
(TIF)

Figure S3 Excessive nitrite exposure diminished expression of valve progenitor makers as early as 43 hpf. Expressions of *vcana* (A, C) and *bmp4* (E, G) were observed in the AVC of control embryos at 43 hpf and 46 hpf. 100 mg/l nitrite exposure from 10 hpf significantly decreased their expressions in the AVC (B, D, F, H), respectively. Expression of *notch1b* was not seen at 43 hpf (I) but initiated at 46 hpf (K) in the AVC of control embryos. 100 mg/l nitrite exposure from 10 hpf abolished *notch1b* expression in the AVC of embryos at 46 hpf (L).
(TIF)

Figure S4 Excessive nitrite exposure did not affect hemodynamics of zebrafish embryos. Embryos were exposed with 100 mg/l nitrite from 10 hpf. After nitrite exposure, embryos derived from *Tg(gata1:DsRed)* exhibited normal development of red blood cells at 24 (A–D), 36 (E–H) and 48 hpf (I–L), respectively. Consistently, *klf2a* did not change its expression pattern at 48 hpf after nitrite exposure (M–N). Similarly, embryos derived from *Tg(flk1:GFP)* displayed normal vessel development at 24 (O-P, O′-P′), 36 (Q-R, Q′-R′) and 48 hpf (S-T, S′-T′), respectively. Panels O′-T′ are the magnification of the region outlined by rectangle in Panel O-T, respectively, showing the normal development of vessels.
(TIF)

Figure S5 Nitrite level was dramatically increased in the nitrite-exposed embryos at 24, 36, 48 and 76 hpf, respectively. The values of nitrite level were shown in Y-axis and the different developmental stages were shown in X-axis. **: P<0.01.
(TIF)

Movie S1 Control zebrafish larvae without nitrite exposure had active responses to mechanical stimuli. Control larvae without nitrite exposure at 76 hpf are able to swim. When receiving mechanical stimuli from a metal needle, they ran away immediately.
(AVI)

Movie S2 Zebrafish larvae exposed with nitrite had no response to mechanical stimuli. Embryos were treated with 100 mg/l nitrite from 10 hpf through 76 hpf. They exhibited no swimming activity. When receiving mechanical stimuli from a metal needle, the nitrite-exposed larvae had no response to the stimuli.
(AVI)

Movie S3 Control embryos without nitrite exposure at 108 hpf had normal blood flow through atria to ventricle.
(AVI)

Movie S4 Embryos exposed with nitrite exhibited blood flow retrograde at 108 hpf. Part of blood cells in the embryos exposed with 100 mg/l nitrite from 10 hpf through 76 hpf flowed back atria from ventricle.
(AVI)

## References

[1] Bryan NS, Loscalzo L (2011) Nitrite and Nitrate in Human Health and Disease. New York: Humana Press. 292p.

[2] Cosby K, Partovi KS, Crawford JH, Patel RP, Reiter CD, et al (2003) Nitrite reduction to nitric oxide by deoxyhemoglobin vasodilates the human circulation. Nat Med 9: 1498–1505. 14595407 10.1038/nm954

[3] Gladwin MT, Crawford JH, Patel RP (2004) The biochemistry of nitric oxide, nitrite, and hemoglobin: role in blood flow regulation. Free Radic Biol Med 36: 707–717. 14990351 10.1016/j.freeradbiomed.2003.11.032

[4] Madigan M, Zuckerbraun B (2013) Therapeutic Potential of the Nitrite-Generated NO Pathway in Vascular Dysfunction. Front Immunol 4: 174. 23847616 10.3389/fimmu.2013.00174 PMC3698458

[5] Gracia R, Shepherd G (2004) Cyanide poisoning and its treatment. Pharmacotherapy 24: 1358–1365. 15628833 10.1592/phco.24.14.1358.43149

[6] McKnight GM, Duncan CW, Leifert C, Golden MH (1999) Dietary nitrate in man: friend or foe? Br J Nutr 81: 349–358. 10615207 10.1017/s000711459900063x

[7] IARC Monographs on the Evaluation of Carcinogenic Risks to Humans (2010) Ingested nitrate and nitrite, and cyanobacterial peptide toxins. IARC Monogr Eval Carcinog Risks Hum 94: : v–vii, 1–412. PMC4781178 21141240

[8] National Primary Drinking Water Regulations: Final Rule, 40. Federal Register (1991) CFR Parts 141–143(56. (20)): 3526–3597.

[9] European Food Safety Authority (2008) Nitrate in vegetables: scientific opinion of the panel on contaminants in the food chain. EFSA J 689: 1–79. 10.2903/j.efsa.2008.653 PMC10193653 37213838

[10] Simmons AE, Karimi I, Talwar M, Simmons TW (2012) Effects of nitrite on development of embryos and early larval stages of the zebrafish (Danio rerio). Zebrafish 9: 200–206. 22823424 10.1089/zeb.2012.0746 PMC3698666

[11] Blue GM, Kirk EP, Sholler GF, Harvey RP, Winlaw DS (2012) Congenital heart disease: current knowledge about causes and inheritance. Med J Aust 197: 155–159. 22860792 10.5694/mja12.10811

[12] Liang D, Zhang M, Bao J, Zhang L, Xu X, et al (2008) Expressions of Raldh3 and Raldh4 during zebrafish early development. Gene Expr Patterns 8(4): 248–253. 18262854 10.1016/j.gep.2007.12.007

[13] Scherz PJ, Huisken J, Sahai-Hernandez P, Stainier DY (2008) High-speed imaging of developing heart valves reveals interplay of morphogenesis and function. Development 135: 1179–1187. 18272595 10.1242/dev.010694

[14] Yutzey KE, Rhee JT, Bader D (1994) Expression of the atrial-specific myosin heavy chain AMHC1 and the establishment of anteroposterior polarity in the developing chicken heart. Development 120: 871–883. 7600964 10.1242/dev.120.4.871

[15] Yelon D, Horne SA, Stainier DY (1999) Restricted expression of cardiac myosin genes reveals regulated aspects of heart tube assembly in zebrafish. Dev Biol 214: 23–37. 10491254 10.1006/dbio.1999.9406

[16] Miquerol L, Gertsenstein M, Harpal K, Rossant J, Nagy A (1999) Multiple developmental roles of VEGF suggested by a LacZ-tagged allele. Dev Biol 212: 307–322. 10433823 10.1006/dbio.1999.9355

[17] Fashena D, Westerfield M (1999) Secondary motoneuron axons localize DM-GRASP on their fasciculated segments. J Comp Neurol 406: 415–424. 10102505 10.1002/(sici)1096-9861(19990412)406:3<415::aid-cne9>3.0.co;2-2

[18] Beis D, Bartman T, Jin SW, Scott IC, D'Amico LA, et al (2005) Genetic and cellular analyses of zebrafish atrioventricular cushion and valve development. Development 132: 4193–4204. 16107477 10.1242/dev.01970

[19] Rottbauer W, Wessels G, Dahme T, Just S, Trano N, et al (2006) Cardiac myosin light chain-2: a novel essential component of thick-myofilament assembly and contractility of the heart. Circ Res 99: 323–331. 16809551 10.1161/01.RES.0000234807.16034.fe

[20] Auman HJ, Coleman H, Riley HE, Olale F, Tsai HJ, et al (2007) Functional modulation of cardiac form through regionally confined cell shape changes. PLoS Biol 5: e53. 17311471 10.1371/journal.pbio.0050053 PMC1802756

[21] Lagendijk AK, Goumans MJ, Burkhard SB, Bakkers J (2011) MicroRNA-23 restricts cardiac valve formation by inhibiting Has2 and extracellular hyaluronic acid production. Circ Res 109: 649–657. 21778427 10.1161/CIRCRESAHA.111.247635

[22] Walsh EC, Stainier DY (2001) UDP-glucose dehydrogenase required for cardiac valve formation in zebrafish. Science 293: 1670–1673. 11533493 10.1126/science.293.5535.1670

[23] Hurlstone AF, Haramis AP, Wienholds E, Begthel H, Korving J, et al (2003) The Wnt/beta-catenin pathway regulates cardiac valve formation. Nature 425: 633–637. 14534590 10.1038/nature02028

[24] Banjo T, Grajcarek J, Yoshino D, Osada H, Miyasaka KY, et al (2013) Haemodynamically dependent valvulogenesis of zebrafish heart is mediated by flow-dependent expression of miR-21. Nat Commun 4: 1978. 23748970 10.1038/ncomms2978 PMC3709480

[25] Vermot J, Forouhar AS, Liebling M, Wu D, Plummer D, et al (2009) Reversing blood flows act through klf2a to ensure normal valvulogenesis in the developing heart. PLoS Biol 7: e1000246. 19924233 10.1371/journal.pbio.1000246 PMC2773122

[26] Jensen FB (2003) Nitrite disrupts multiple physiological functions in aquatic animals. Comp Biochem Physiol A Mol Integr Physiol 135: 9–24. 12727546 10.1016/s1095-6433(02)00323-9

[27] Jensen FB (2007) Nitric oxide formation from nitrite in zebrafish. J Exp Biol 210: 3387–3394. 17872992 10.1242/jeb.008748

[28] Bryan NS, Fernandez BO, Bauer SM, Garcia-Saura MF, Milsom AB, et al (2005) Nitrite is a signaling molecule and regulator of gene expression in mammalian tissues. Nat Chem Biol 1: 290–297. 16408059 10.1038/nchembio734

[29] Brunner F, Stessel H, Kukovetz WR (1995) Novel guanylyl cyclase inhibitor, ODQ reveals role of nitric oxide, but not of cyclic GMP in endothelin-1 secretion. FEBS Lett 376: 262–266. 7498555 10.1016/0014-5793(95)01297-x

[30] Staudt D, Stainier D (2012) Uncovering the molecular and cellular mechanisms of heart development using the zebrafish. Annu Rev Genet 46: 397–418. 22974299 10.1146/annurev-genet-110711-155646 PMC6982417

[31] Ataya B, Tzeng E, Zuckerbraun BS (2011) Nitrite-generated nitric oxide to protect against intimal hyperplasia formation. Trends Cardiovasc Med 21: 157–162. 22814422 10.1016/j.tcm.2012.05.002

[32] Bryan NS, Calvert JW, Gundewar S, Lefer DJ (2008) Dietary nitrite restores NO homeostasis and is cardioprotective in endothelial nitric oxide synthase-deficient mice. Free Radic Biol Med 45: 468–474. 18501719 10.1016/j.freeradbiomed.2008.04.040 PMC2662396

[33] Lundberg JO, Gladwin MT, Ahluwalia A, Benjamin N, Bryan NS, et al (2009) Nitrate and nitrite in biology, nutrition and therapeutics. Nat Chem Biol 5: 865–869. 19915529 10.1038/nchembio.260 PMC4038383

[34] Hunter L, Gordge L, Dargan PI, Wood DM (2011) Methaemoglobinaemia associated with the use of cocaine and volatile nitrites as recreational drugs: a review. Br J Clin Pharmacol 72: 18–26. 21352269 10.1111/j.1365-2125.2011.03950.x PMC3141183

[35] Combs MD, Yutzey KE (2009) Heart valve development: regulatory networks in development and disease. Circ Res 105: 408–421. 19713546 10.1161/CIRCRESAHA.109.201566 PMC2777683

[36] Rastaldo R, Pagliaro P, Cappello S, Penna C, Mancardi D, et al (2007) Nitric oxide and cardiac function. Life Sci 81: 779–793. 17707439 10.1016/j.lfs.2007.07.019

[37] Chang AC, Fu Y, Garside VC, Niessen K, Chang L, et al (2011) Notch initiates the endothelial-to-mesenchymal transition in the atrioventricular canal through autocrine activation of soluble guanylyl cyclase. Dev Cell 21: 288–300. 21839921 10.1016/j.devcel.2011.06.022

[38] Feng Q, Song W, Lu X, Hamilton JA, Lei M, et al (2002) Development of heart failure and congenital septal defects in mice lacking endothelial nitric oxide synthase. Circulation 106: 873–879. 12176963 10.1161/01.cir.0000024114.82981.ea

[39] Haddad EK, Duclos AJ, Baines MG (1995) Early embryo loss is associated with local production of nitric oxide by decidual mononuclear cells. J Exp Med 182: 1143–1151. 7561687 10.1084/jem.182.4.1143 PMC2192282

[40] Garg V, Muth AN, Ransom JF, Schluterman MK, Barnes R, et al (2005) Mutations in NOTCH1 cause aortic valve disease. Nature 437: 270–274. 16025100 10.1038/nature03940

[41] Balashova N, Chang FJ, Lamothe M, Sun Q, Beuve A (2005) Characterization of a novel type of endogenous activator of soluble guanylyl cyclase. J Biol Chem 280(3): 2186–2196. 15509556 10.1074/jbc.M411545200

[42] Marshall HE, Merchant K, Stamler JS (2000) Nitrosation and oxidation in the regulation of gene expression. FASEB J 14(13): 1889–1900. 11023973 10.1096/fj.00.011rev

