# Correction: Excessive Nitrite Affects Zebrafish Valvulogenesis through Yielding Too Much NO Signaling

**DOI:** 10.1371/journal.pone.0115217

**Published:** 2014-12-02

**Authors:** The PLOS ONE Staff

There is an error in the Materials and Methods in the last sentence of the first paragraph under the subheading “Whole mount *in situ* hybridizations.” The primer sequences for *vmhc* are incorrect. The primer sequences for *vmhc* should read “F: AAAGA CTCCT GGTGC AATG” and “R: TTCAG CTCAG AGCAT TCGT.”
