# Supplementary figures and images for: Excessive Nitrite Affects Zebrafish Valvulogenesis through Yielding Too Much NO Signaling
Source: PLoS One. 2014 Mar 21;9(3):e92728. doi: 10.1371/journal.pone.0092728 (PMC3962429; doi:10.1371/journal.pone.0092728)

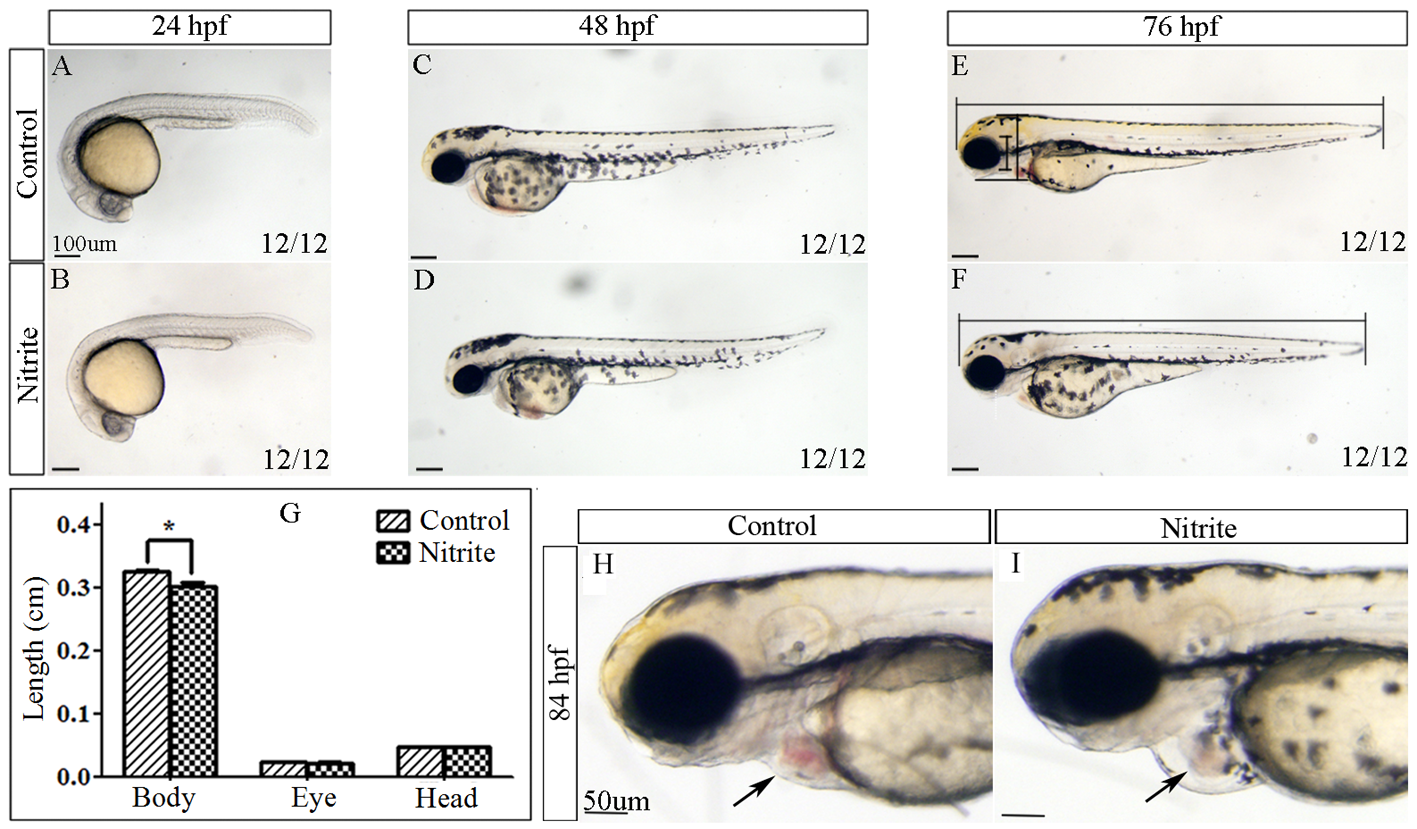

Supplement: Figure S1 — Morphological changes of nitrite-exposed embryos at early development. Nitrite-exposed embryos were treated with 100 mg/l sodium nitrite from 10 hpf. They displayed a similar morphological phenotype to control embryos at 24 hpf (A, B). At 48 hpf, the nitrite-exposed embryos (D) looked very similar to control embryos (C) except that they showed a slightly shortened body length (D). When reaching 76 hpf, the nitrite-exposed embryos displayed normal diameter of eye, and heart development (E, G) when compared to control embryos (F, G). However, the body length of the exposed embryos was significantly shorter than control embryos (F, G). At 84 hpf, some of nitrite-exposed embryos started to exhibit cardiac edema (H, I). *, P<0.05. (TIF) [file pone.0092728.s001.tif]

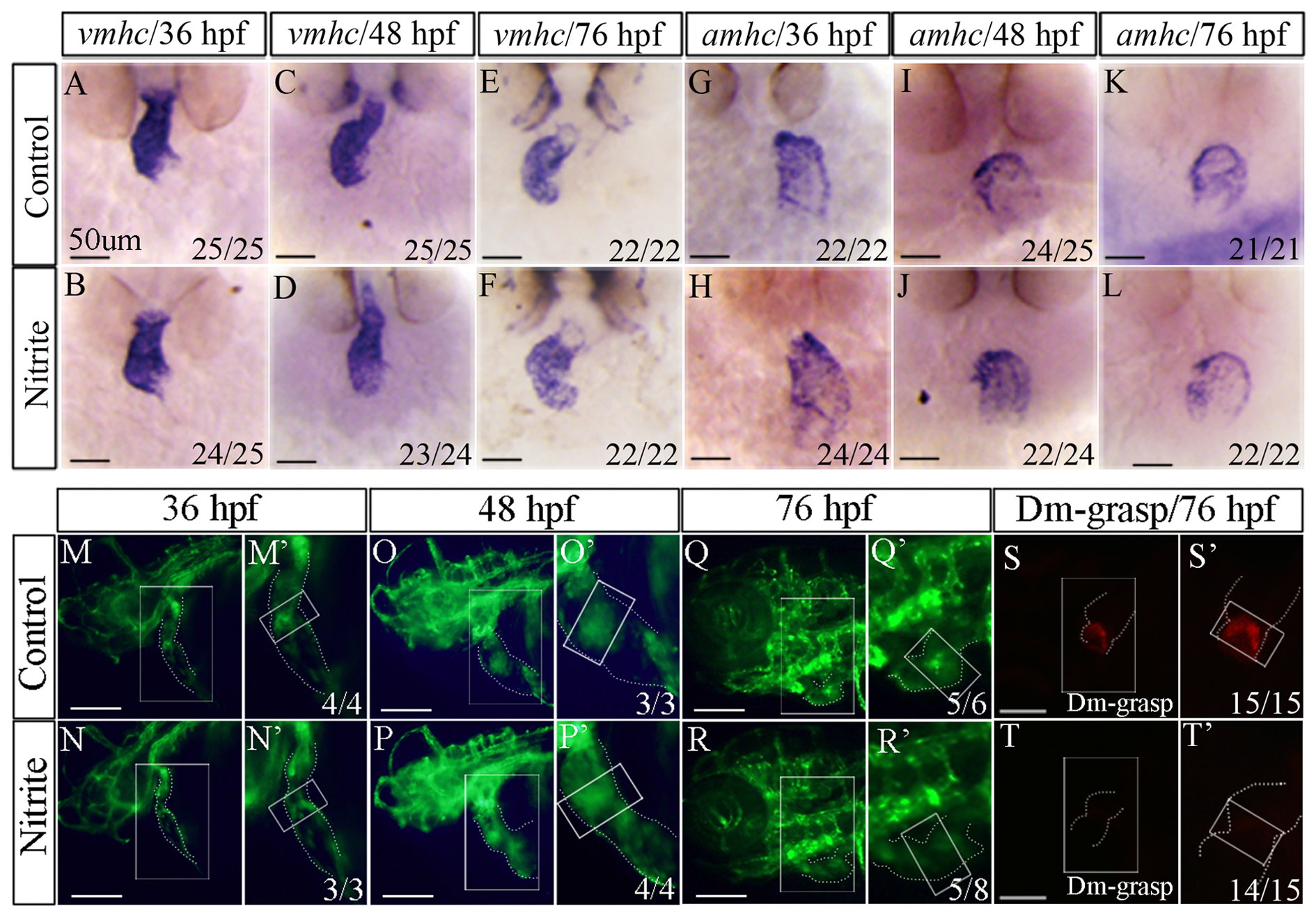

Supplement: Figure S2 — Excessive nitrite exposure affected endothelial cell accumulation and differentiation in the AVC at 76 hpf. Nitrite-exposed embryos were treated with 100 mg/l sodium nitrite from 10 hpf. Expression patterns of vmhc in ventricle and amhc in atria of nitrite-exposed embryos were similar to those in control embryos at 36 (A, B; G, H), 48 (C, D; I, J) and 76 hpf (E, F; K, L), respectively. Excessive nitrite exposure did not change the distribution of endothelial cell in the AVC of embryos at 36 hpf (M-N, M′-N′) and 48 hpf (O-P, O′-P′). Compared to control embryos at 76 hpf (Q-S; Q′-S′), 5/8 nitrite-treated embryos had fewer endothelial cells (R, R′) and 14/15 nitrite-exposed embryos lost the expression of Dm-grasp (T, T′), maker of endothelial cell differentiation in AVC, at the same stage. Panel M′-T′ were the magnification of the region outlined by rectangle in Panel M-T, respectively. Heart was outlined by dot-lined curves and the AVC in Panel M′-T′ was outlined by a small rectangle. (TIF) [file pone.0092728.s002.tif]

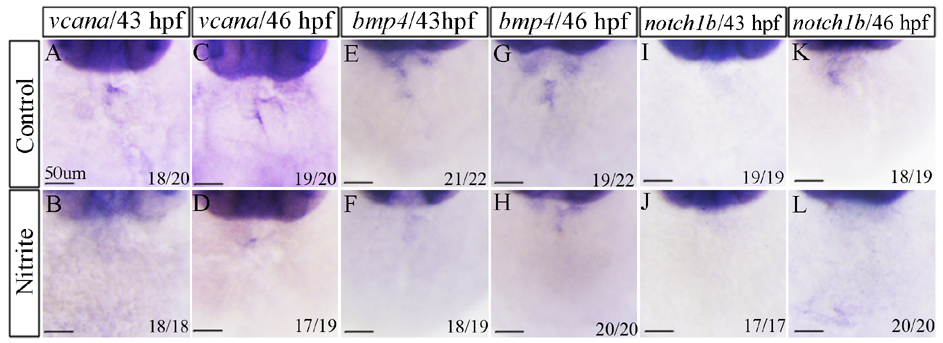

Supplement: Figure S3 — Excessive nitrite exposure diminished expression of valve progenitor makers as early as 43 hpf. Expressions of vcana (A, C) and bmp4 (E, G) were observed in the AVC of control embryos at 43 hpf and 46 hpf. 100 mg/l nitrite exposure from 10 hpf significantly decreased their expressions in the AVC (B, D, F, H), respectively. Expression of notch1b was not seen at 43 hpf (I) but initiated at 46 hpf (K) in the AVC of control embryos. 100 mg/l nitrite exposure from 10 hpf abolished notch1b expression in the AVC of embryos at 46 hpf (L). (TIF) [file pone.0092728.s003.tif]

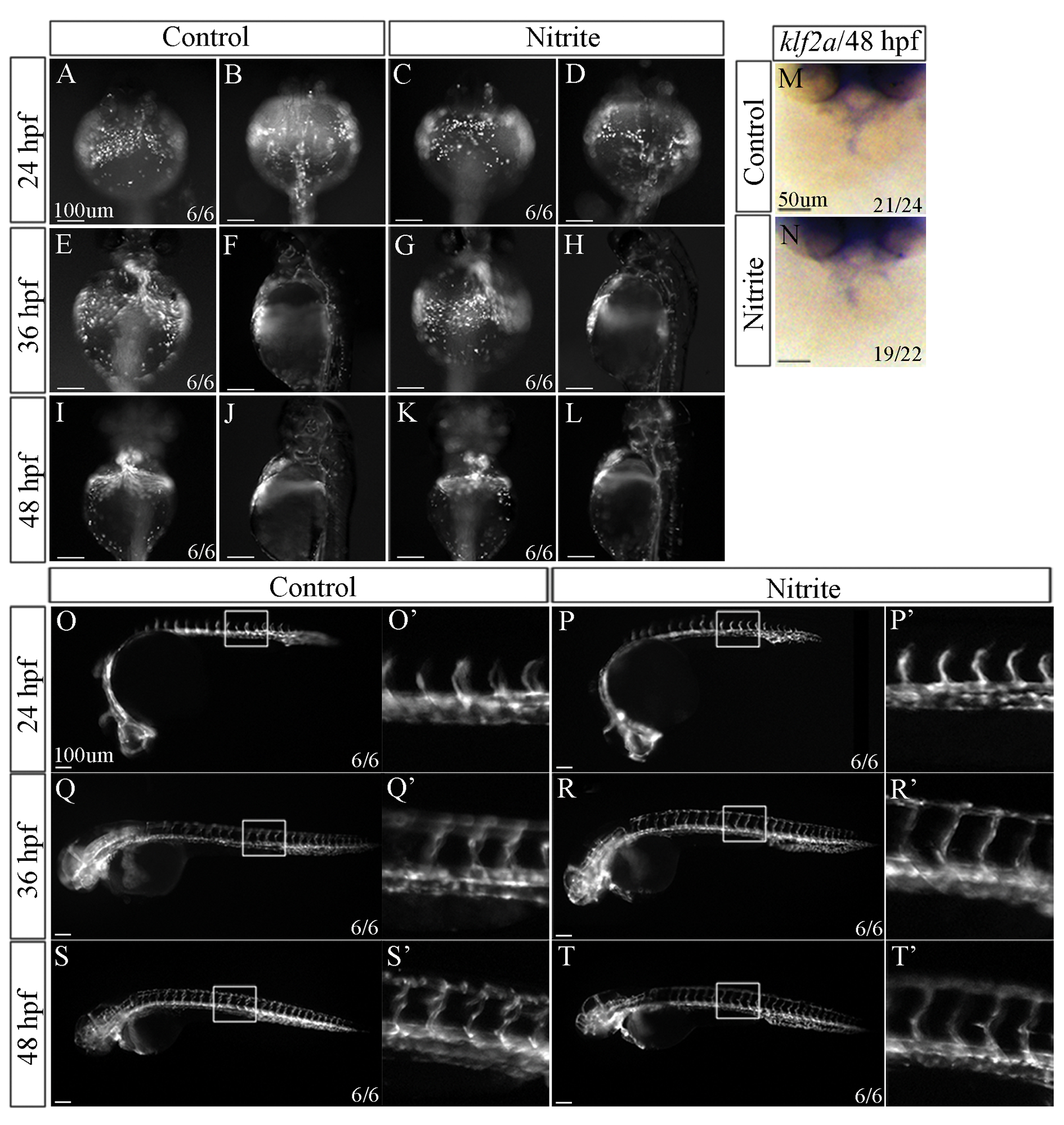

Supplement: Figure S4 — Excessive nitrite exposure did not affect hemodynamics of zebrafish embryos. Embryos were exposed with 100 mg/l nitrite from 10 hpf. After nitrite exposure, embryos derived from Tg(gata1:DsRed) exhibited normal development of red blood cells at 24 (A–D), 36 (E–H) and 48 hpf (I–L), respectively. Consistently, klf2a did not change its expression pattern at 48 hpf after nitrite exposure (M–N). Similarly, embryos derived from Tg(flk1:GFP) displayed normal vessel development at 24 (O-P, O′-P′), 36 (Q-R, Q′-R′) and 48 hpf (S-T, S′-T′), respectively. Panels O′-T′ are the magnification of the region outlined by rectangle in Panel O-T, respectively, showing the normal development of vessels. (TIF) [file pone.0092728.s004.tif]

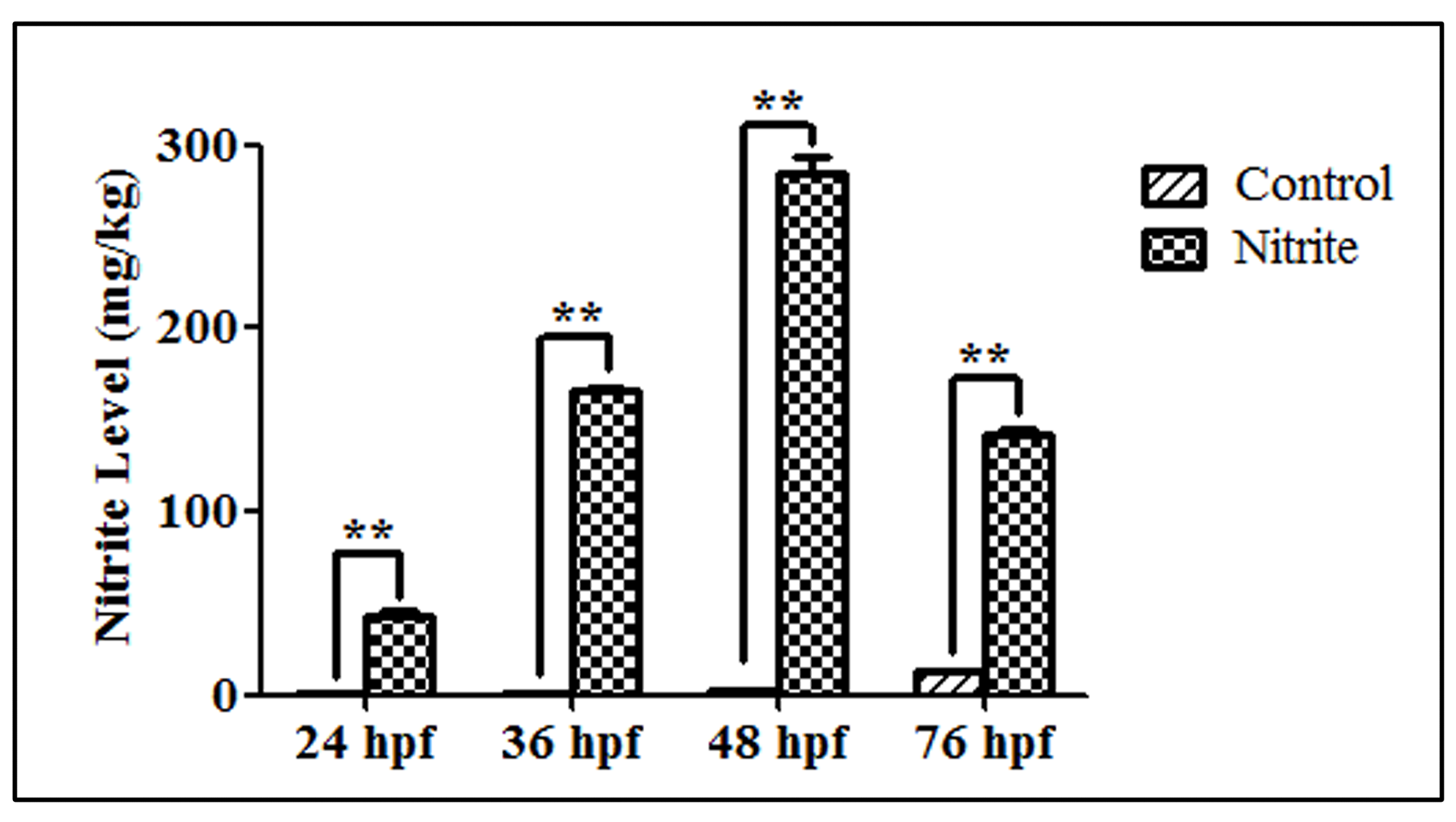

Supplement: Figure S5 — Nitrite level was dramatically increased in the nitrite-exposed embryos at 24, 36, 48 and 76 hpf, respectively. The values of nitrite level were shown in Y-axis and the different developmental stages were shown in X-axis. **: P<0.01. (TIF) [file pone.0092728.s005.tif]
